# Enhancing the implementation of the Making Every Contact Count brief behavioural intervention programme in Ireland: protocol for the Making MECC Work research programme

**DOI:** 10.12688/hrbopenres.13481.1

**Published:** 2022-01-18

**Authors:** Oonagh Meade, Maria O'Brien, Jenny Mc Sharry, Agatha Lawless, Sandra Coughlan, Jo Hart, Catherine Hayes, Chris Keyworth, Kim L Lavoie, Andrew W Murphy, Patrick Murphy, Chris Noone, Orlaith O'Reilly, Health Psychology Public Advisory Panel, Molly Byrne

**Affiliations:** 1 Health Behaviour Change Research Group, School of Psychology, NUI Galway, Galway, H91 EV56, Ireland; 2 National Heart Programme, Integrated Care Programme for Chronic Disease, Clinical Design and Innovation, Office of the Chief Clinical Officer, Health Services Executive, Áras Sláinte, Wilton Road, Cork, T12 XRR0, Ireland; 3 Health & Wellbeing, Strategy and Research, Healthcare Strategy, c/o Health Promotion and Improvement Office, Health Service Executive, Waterford, X91 T256, Ireland; 4 Strategic Planning and Transformation, Health Service Executive, Cork, T12 WP62, Ireland; 5 School of Medical Sciences, University of Manchester, Manchester, M13 9PT, UK; 6 Public Health and Primary Care, School of Medicine, Trinity College Dubin, Dublin, D02 R590, Ireland; 7 School of Psychology, University of Leeds, Leeds, LS2 9JT, UK; 8 Montréal Behavioural Medicine Centre, CIUSSS-NIM, Hôpital Sacré-Cœur de Montréal, Montréal, Quebec, QC H4J 1C5, Canada; 9 Department of Psychology, University of Quebec at Montréal, Montréal, Quebec, QC H2L 2C4, Canada; 10 Health Research Board Primary Care Clinical Trials Network Ireland, School of Medicine, NUI Galway, Galway, Ireland; 11 Health and Wellbeing Division, HSE South East, Public Health Department, Health Service Executive, Kilkenny, Ireland

**Keywords:** Making Every Contact Count, chronic illness prevention, brief behavioural intervention, smoking, diet, exercise, alcohol and drug use, implementation strategy

## Abstract

**Background:** Brief behavioural interventions offered by healthcare professionals to target health behavioural risk factors (e.g. physical activity, diet, smoking and drug and alcohol use) can positively impact patient health outcomes. The Irish Health Service Executive (HSE) Making Every Contact Count (MECC) Programme supports healthcare professionals to offer patients brief opportunistic behavioural interventions during routine consultations. The potential for MECC to impact public health depends on its uptake and implementation.

**Aim:** This protocol outlines the ‘Making MECC Work’ research programme, a HSE/Health Behaviour Change Research Group collaboration to develop an implementation strategy to optimise uptake of MECC in Ireland. The programme will answer three research questions:

(1) What determines delivery of MECC brief interventions by healthcare professionals at individual and organisational levels?

(2) What are patient attitudes towards, and experiences of, receiving MECC interventions from healthcare professionals?

(3) What evidence-informed implementation strategy options can be consensually developed with key stakeholders to optimise MECC implementation?

**Methods: **In Work Package 1, we will examine determinants of MECC delivery by healthcare professionals using a multi-methods approach, including: (WP1.1) a national survey of healthcare professionals who have participated in MECC eLearning training and (WP1.2) a qualitative interview study with relevant healthcare professionals and HSE staff. In Work Package 2, we will examine patient attitudes towards, and experiences of, MECC using qualitative interviews. Work Package 3 will combine findings from Work Packages 1 and 2 using the Behaviour Change Wheel to identify and develop testable implementation strategy options (WP 3.1). Strategies will be refined and prioritised using a key stakeholder consensus process to develop a collaborative implementation blueprint to optimise and scale-up MECC (WP3.2).

**Discussion:** Research programme outputs are expected to positively support the integration of MECC brief behaviour change interventions into the Irish healthcare system and inform the scale-up of behaviour change interventions internationally.

## Introduction

Rapidly increasing rates of chronic disease are a key global societal challenge. Chronic diseases, such as cardiovascular diseases, diabetes, cancer and chronic respiratory diseases, are the leading global cause of disability and are responsible for 70% of deaths worldwide ( World Health Organization, 2017). It has been estimated that in Ireland approximately one million people live with heart disease, diabetes or respiratory disease ( Chronic Conditions Working Group, 2017) and the prevalence of chronic conditions is increasing over time ( McNicholas & Laird, 2018). In Ireland, approximately 80% of GP consultations ( Department of Health and Children, 2008) and 76% of hospital bed days are used by patients with chronic conditions ( Jennings, 2014). Chronic diseases in Ireland are associated with 86% of mortality and 77% of the overall disease burden and patients with chronic diseases presently utilise around 70% of health resources ( Department of Health, 2012). This burden of chronic disease is likely to increase over time due to aging populations in Ireland, and internationally.

Changing health-related behaviours (e.g. smoking, poor diet, excessive alcohol consumption and physical inactivity) has the potential to significantly impact on patterns of chronic disease and reduce leading causes of mortality and morbidity ( National Institute for Health and Clinical Excellence, 2007). However, current rates of engagement in health-related behaviours are sub-optimal; 17% of the Irish population are current smokers and 46% are achieving the minimum recommended physical activity guidelines ( Department of Health, 2019), 34% consume unhealthy foods daily and 37% of drinkers binge drink on a typical occasion ( Department of Health, 2018).

Systematic review evidence suggests that brief behavioural interventions delivered by healthcare professionals can impact positively on smoking ( Stead *et al.*, 2013), physical activity ( Lamming *et al.*, 2017), dietary behaviours ( Whatnall *et al.*, 2018), alcohol consumption ( Kaner *et al.*, 2018) and drug use ( Lynch *et al.*, 2020). There is also evidence to suggest that such interventions are cost effective ( National Institute for Health and Clinical Excellence, 2014). In light of the promising evidence of brief interventions for behaviour change, the Irish Making Every Contact Count (MECC) programme, a national health behaviour change programme, was initiated by the Irish Health Service Executive (HSE) in 2017. The MECC programme supports the implementation of Healthy Ireland, a government-led programme which aims to encourage and support the physical and mental health of people living in Ireland ( Department of Health, 2013). It also complements the National Self-management Support Framework for Chronic Conditions: COPD, Asthma, Diabetes and Cardiovascular disease ( Chronic Conditions Working Group, 2017) through its focus on training staff to support people to self-manage chronic conditions. Specifically, the MECC programme in Ireland is designed to enable healthcare professionals to use brief behavioural interventions in routine healthcare consultations to support patients in making health behaviour changes in relation to smoking, alcohol and drug use, physical activity and healthy eating ( Health Service Executive, 2016).

Feedback from public health practitioners who had a role in supporting implementation of a Making Every Contact Count policy in the UK suggests that standardisation of training for healthcare professionals may enhance MECC implementation ( Chisholm *et al.*, 2019). The MECC training programme is an essential component of the implementation of MECC in the Irish health service. Standardised online training curricula and face-to-face training courses have been developed for healthcare professionals to enable them to initiate conversations about health behavioural risk factors, and to deliver brief interventions. Training for healthcare professionals involves completion of an eLearning training programme which contains six modules. These include an introduction to behaviour change theory and techniques and taking a patient-centred approach; four topic modules on smoking, alcohol and drugs, healthy eating and active living; and a final skills into practice module which contains a series of video demonstrations by healthcare professionals on how to conduct brief interventions. Those who complete the eLearning programme have the option of joining a half-day skills development workshop where they have the opportunity to role-play brief intervention delivery with their peers and receive feedback from programme trainers. In parallel to the training for healthcare professionals, a standardised curriculum has also been developed for undergraduate and postgraduate healthcare students and is being implemented in third level institutions across Ireland.

The MECC training programme adopts the ‘5As approach’ to brief interventions. This is a flexible framework to help healthcare professionals have a conversation with patients about health behaviour change, originally developed for smoking cessation ( Fiore *et al.*, 2008) and adapted for use in other areas (e.g. obesity, Vallis *et al.*, 2013). The 5As framework involves the following steps: ask about the behaviour; advise on the need for behaviour change; assess readiness to change; assist with exploring benefits and barriers of change, identifying options for change and goal setting; and arrange referral to more intensive support if appropriate.

The MECC Framework ( Health Service Executive, 2016) sets out the vision and objectives of the MECC programme and an implementation plan for the Irish Health Service. This includes key actions to implement the MECC programme across frontline services in Community and Acute settings in Ireland. In the initial phases of MECC implementation in Ireland, which started in 2019, approximately 60 exemplar healthcare sites were selected for roll-out of the programme. Implementation is supported by the national MECC team and local Health Promotion and Improvement staff who support Hospital Groups and Community Health Organisations to work through the six phases of implementation: mapping and buy-in with senior management, identification of sites, planning for implementation at site level, staff training, onsite implementation and monitoring and review of progress.

The ability of MECC to reach its potential and positively impact on public health is dependent on its successful and widespread uptake and implementation. A systematic review of systematic reviews recently narratively synthesised barriers and enablers to the delivery of brief behaviour change interventions across healthcare professional groups internationally ( Keyworth *et al.*, 2020b). Perceptions of their knowledge and skills to deliver interventions, perceptions of their healthcare professional role, beliefs about resources and support needed, and healthcare professionals’ own health behaviour acted as both barriers and enablers to delivery of behaviour change interventions. Common additional barriers included lack of time, a perceived lack of prioritisation of behaviour change interventions, negative attitudes towards patients and perceptions of patients’ risk and of patients’ motivation. Three common enablers identified were training, context and professionals’ attitudes towards delivery of such interventions.

A number of systematic reviews of the delivery of brief behaviour change interventions by specific types of healthcare professionals about specific health behaviours or to patients with specific health conditions highlight additional complex barriers and enablers to brief intervention delivery. For example, a review of qualitative literature of doctors’ views and experiences of delivering weight loss interventions within routine consultations, identified additional barriers including: pessimism about patients’ weight loss success, feelings of hopelessness and frustration, and the nature of the physician-patient relationship ( Dewhurst *et al.*, 2017). A systematic review of the delivery of smoking cessation interventions by oncology health practitioners revealed that in addition to skills, knowledge and workplace procedures influencing delivery of smoking interventions, the perception of benefit to patients was also an important factor in brief intervention delivery ( Conlon *et al.*, 2017). A review of the provision of brief interventions for alcohol reduction suggests that the context of delivery is important with professionals and patients reporting that that well-being clinics and check-ups are a more acceptable opportunity for delivery rather than during consultations focused on different health conditions ( Johnson *et al.*, 2011).

Evidence from the UK MECC initiative indicates that, while healthcare professionals do appreciate the ‘intuitive’ nature of the programme and value its simplicity, evidence-based nature and fit with routine practice, take-up across different parts of the health service is varied ( Nelson *et al.*, 2013). A survey of UK healthcare professionals’ engagement with MECC policy indicates only one third of healthcare professionals had heard of the framework, and only half of healthcare professionals deliver interventions when they perceived that patients would benefit from them ( Keyworth *et al.*, 2018). While healthcare professionals across different professional groups see the value in delivering brief opportunistic interventions, they are sceptical about their capability to do so and can be constrained by their working environments ( Keyworth *et al.*, 2019).

For MECC to succeed, not only do healthcare professionals need to adopt it in practice, but patients need to find such interventions by healthcare professionals acceptable and supportive. There is evidence to suggest that healthcare professionals’ willingness to offer brief behavioural interventions is influenced by their perceptions of how receptive patients might be to such conversations ( Bonner *et al.*, 2015; Stead *et al.*, 2009). Research suggests that patients in certain contexts are open to discussing behaviour change when attending routine healthcare consultations. Patients sometimes want and expect health care professionals to start these conversations ( Keyworth *et al.*, 2020a; Nelson *et al.*, 2016). For example, Aveyard *et al.* (2016) found that a brief opportunistic weight loss intervention delivered by general practitioners was acceptable to patients; over 80% of patients reported that the intervention was appropriate and helpful. In addition, 64% of smokers and recent quitters reported willingness to receive brief behavioural interventions at lung screening appointments ( Stevens *et al.*, 2019). In a recent qualitative study, GPs were found to be an appropriate and credible source of brief behaviour change interventions as they are a key (and often first) point of contact for patients in the health service and rapport between patients and GPs could be a key facilitator of the use of such interventions ( Keyworth *et al.*, 2020a). While patient acceptability has been demonstrated for specific target behaviours in specific healthcare contexts, patients’ experiences of, and preferences for, national programmes targeting multiple behavioural targets, delivered by different types of healthcare professionals are underexplored. The patient perspective is an important element in the implementation of MECC; if patients report finding these types of initiatives acceptable and helpful, this information has the potential to act as an important lever of healthcare professional behaviour change.

There is little international literature currently on the implementation of behaviour change programmes such as MECC which take a standardised national approach to brief intervention training and implementation support. The proposed programme has the potential to add uniquely to international knowledge in this area. The protocol below will outline the Making MECC Work research programme developed by the HSE MECC team and the Health Behaviour Change Research Group (HBCRG) at NUI Galway which aims to develop a collaborative implementation strategy for optimising MECC, to embed the routine use of brief behaviour change interventions by healthcare professionals.

## Methods

**Figure 1.**
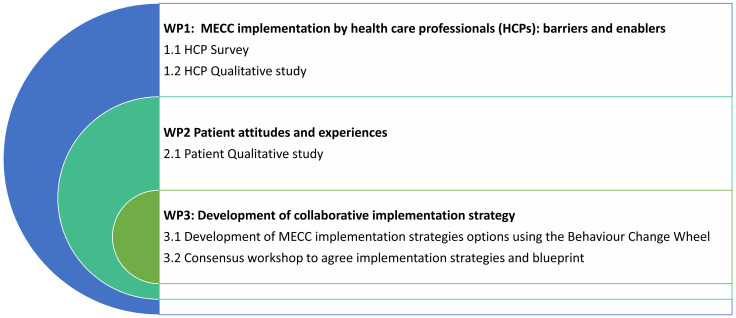
Visual model of the study work packages.

The research programme consists of three related work packages (WPs). We will examine healthcare professional-level and organisational-level barriers and enablers to implementing MECC quantitatively and qualitatively (WP1). Patient experiences of receipt of MECC will be explored qualitatively (WP2). We will integrate our findings using an established implementation intervention development process and a stakeholder consensus workshop to generate a collaborative implementation strategy for MECC (WP3). The protocol for the five empirical studies (studies 1.1, 1.2, 2.1, 3.1 and 3.2) is described in detail below. A visual model of the three WPs is presented in Figure 1. The research programme is registered with the Open Science Framework ( Meade *et al.*, 2021).

### Integrated knowledge translation approach

This research programme was developed to directly respond to the evidence needs of the HSE MECC programme team. The study is jointly led by an academic Principal Investigator (MB) and knowledge user Principal Investigator (MO’B). The Study Management Team which oversees operational aspects of the research programme contains both academic and knowledge user members. The Study Steering Committee which reviews the progress of the study against the agreed schedule, the achievement of agreed deliverables, and spending against the approved budgetary allocations also consists of academic and HSE knowledge user co-applicants and collaborators on the research programme. The combined knowledge user-academic governance structure aims to enhance the delivery of the studies and the relevance and uptake of any study recommendations.

Members of the Study Management Team will attend quarterly meetings of the national HSE MECC Implementation Group (national senior HSE representatives charged with overseeing the implementation of the MECC programme), to update the group of study findings and ensure that the study is grounded in the evidence requirements of the group. We will seek advice on issues including recruitment methods, the acceptability and feasibility of proposed data collection methods and tools, participant documentation and optimal methods for dissemination that will maximise the translational impact of the research programme.

To ensure our research and resulting implementation strategy are grounded in those who use health services, the ‘Health Psychology Public Advisory Panel’ a seven-member Patient and Public Involvement (PPI) panel has been established at NUI Galway to support the research programme. PPI contributors support the team on key tasks including the development of recruitment strategies, identifying and refining questions for use in interviews/survey, advising on patient documentation, and contributing to data analysis. We hold eight meetings per year online/face-to-face and provide flexible opportunities for involvement by e-mail/phone/post. PPI contributors are provided with any training and supports needed to complete PPI tasks and are compensated for their time.

### WP1 – MECC implementation by healthcare professionals: barriers and enablers

**Work package overview**

WP1 will focus on understanding barriers and enablers to implementing MECC from the perspective of healthcare professionals and staff involved in supporting implementation of MECC. A national survey (study 1.1) will be conducted with healthcare professionals to identify barriers and enablers to delivering MECC brief interventions. A qualitative study (study 1.2) will be conducted with clinical and non-clinical staff who have a role in implementing MECC within HSE sites that had varied levels of success in implementing MECC in order to understand individual and organisational-level barriers and enablers to MECC implementation in greater depth.

**Study 1.1 National survey of healthcare professionals who have completed MECC eLearning training**

***Aims***

 • To identify and quantify individual-level and organisational-level barriers and enablers to the implementation of MECC in routine healthcare from the perspective of healthcare professionals.

• To examine relationships between potentially modifiable barriers and enablers to MECC implementation and healthcare professionals' delivery of MECC interventions.

***Design***

A cross-sectional online survey will be used to examine individual and organisational-level barriers and enablers to MECC delivery by healthcare professionals.

***Methods***

All healthcare professionals who have completed the online MECC eLearning training programme from 2018–2021 (n=4050) will be invited to participate in the online survey via an e-mail invitation from the HSE MECC team.

The survey will be hosted via www.qualtrics.com, a GDPR compliant online survey tool. The e-mail invitation to the survey will be distributed by the HSE MECC team and will be open for six weeks. A reminder will be sent to healthcare professionals two weeks after the initial invitation. Healthcare professionals will be offered the opportunity to enter a prize draw for one of four €50 shopping vouchers as an incentive to participate.

In brief, our survey will consist of four sections: demographic information; MECC training uptake; use of MECC interventions; and barriers and enablers to the use of MECC. A copy of the survey is publicly available via the Open Science Framework ( Meade *et al.*, 2021).

*1. Demographic information*

Demographic details which will be requested include: age, gender, professional role, workplace setting, years qualified and work contract (full-time/part time).

*2. MECC training uptake*

MECC training uptake questions will include what year they completed the eLearning module, whether they have completed the ‘Enhancing your Brief Intervention Skills’ face-to-face training and two five point Likert-style rating scales of their satisfaction with the face-to-face training and the likelihood that they would recommend the course to others.

*3. Use of MECC interventions*

The primary outcome measure to assess level of use of MECC interventions will be a single question asking participants if they have ever delivered a MECC intervention (yes/no). Participants will also be asked what proportion of their weekly patients it would be appropriate to deliver a MECC intervention to, what proportion of eligible patients they deliver MECC interventions to, and what health behaviours they have addressed in any MECC interventions delivered. Participants will be asked about how frequently they document (record) MECC interventions and how easy it is to document these on five point Likert scales. They will also be asked where they document such interventions and an open-ended comment box will be provided so they can describe what makes it easy or difficult to document MECC interventions.

Skip logic will be used throughout the survey to ensure that participants are directed to relevant questions based on their responses to questions. For example, those who have not ever delivered a MECC intervention will not be asked questions about recording MECC interventions.

*4. Barriers and enablers to the use of MECC*

Five questions will be asked about the impact of COVD-19 on MECC brief intervention delivery. These will ask about the impact of COVID-19 on opportunities for MECC delivery, the difficulty of delivering MECC interventions, participants’ comfort with delivering MECC interventions, participants’ ability to prioritise MECC, and their ability to deliver MECC in phone/online consultations. These items will be combined to create an outcome ‘impact of COVID-19 on MECC delivery’.

The next section of the survey contains 44 five-point Likert scale items designed to address potentially modifiable barriers and enablers to MECC brief intervention delivery. The items are informed by the Theoretical Domains Framework (TDF), a comprehensive framework of determinants of behaviour originally developed to understand influences on healthcare professionals’ behaviour ( Cane *et al.*, 2012; Michie *et al.*, 2005). Items from an existing TDF survey of implementation behavioural determinants ( Huijg *et al.*, 2014) have been adapted to the MECC context to gather information on healthcare professionals’ experiences of barriers and enablers to the delivering MECC brief interventions. The items were refined through consultations with the Study Management Team, the Study Steering Committee, the MECC Implementation Group, and the Health Psychology Public Advisory Panel. The domains of the TDF that will be measured are: knowledge; skills; social/professional role identity; beliefs about capabilities; optimism; beliefs about consequences; reinforcement; intentions; goals; memory, attention and decision processes; environmental context and resources; social influences; emotions; and behavioural regulation. We will use a confirmatory factor analysis to test one-factor solutions for each domain scale. Items with factor loadings lower than .3 will be removed from domain scales. These one-factor solutions will be retained if the model fit is adequate. Specifically, if the Root Mean Square Error of Approximation is less than 0.10, the one-factor solution will be accepted, and if it is greater than 0.10, we will assume that a one-factor solution does not fit the items and exploratory factor analysis will be used to identify an optimal solution. The internal consistency of each scale will be assessed using McDonald’s Omega through a SPSS macro ( Hayes & Coutts, 2020)

Finally, participants will also be asked to document in open text boxes the top three enablers of delivering MECC brief interventions and top three barriers. At the end of the survey we will invite survey participants to consent to be contacted about participating in a follow-up qualitative interview (study 1.2) to discuss their experiences of enablers and barriers to the delivery of MECC interventions in greater depth.

***Data analysis***

We will use descriptive statistics to describe demographics, MECC training uptake, use of MECC interventions and barriers and enablers identified to the delivery of MECC brief interventions by healthcare professionals. Logistic regression analysis will be used to examine the relationships between modifiable barriers and enablers to MECC implementation as measured using our adapted TDF questionnaire and healthcare professionals’ delivery of MECC interventions (primary outcome). In the regression analysis we will control for the effects of non-modifiable determinants of MECC delivery (demographic variables and the impact of COVID-19 on MECC delivery). A CIBER analysis will also be conducted to examine the univariate relationship between each predictor and our primary outcome in order to understand the relative importance of each determinant. As an exploratory analysis, we will also conduct a linear regression to examine the relationship between the proportion of times healthcare professionals deliver MECC interventions to eligible patients and modifiable barriers and enablers to MECC delivery as measured using our adapted TDF questionnaire. The outcome measure in this linear regression will be a one item measure of the proportion of times participants deliver a MECC intervention to their patients when they feel it is appropriate. Open ended responses on barriers/enablers to MECC delivery will be coded using a Framework Analysis approach ( Gale *et al.*, 2013) guided by the TDF framework.

***Sample size***

Recent online surveys of HSE healthcare professionals have resulted in response rates of between 5–15%. For this survey, we will aim to reach a response rate of 10% (n=405) of our total population of healthcare professionals who have completed MECC training (n=4050). Previous research in the UK indicated that 50% of healthcare professionals reported delivering MECC interventions where they perceived patients would benefit from them ( Keyworth *et al.*, 2018). In calculating our sample size, we estimated that a greater percentage of our sample (80%) will have delivered a MECC intervention as all participants will have engaged in MECC eLearning training so may be more motivated/prepared to deliver MECC interventions.

If we reach our recruitment target, we will be powered to detect an odds ratio of at least 1.66 in our primary logistic regression analysis. This was the most conservative estimate determined through an a priori power analysis conducted with G*Power 3.1 ( Faul *et al.*, 2009). We aimed for 80% power and set an alpha level of .05 with one tail as it was not feasible to set a higher power or lower alpha given the likely response rate and we expected the predictors to be associated with greater odds of delivering a MECC intervention. We assumed that the predictors would be normally distributed with a mean of 2 and a standard deviation of 1, as the items were 5-point Likert scales. We varied the R ^2 ^between the predictors from .04 to .25, reflecting weak to moderate correlations, to examine differences in the required sample size across a range of minimum effect sizes. These analyses suggested that, given the assumptions described above, a moderate or stronger odds ratio can be detected with our predicted sample size. Sample size calculation information is available via the Open Science Framework ( Meade *et al.*, 2021).

**Study 1.2 Qualitative study of barriers and enablers of MECC implementation**

***Aim***

To gain an in-depth understanding of the individual-level and organisational-level enablers of and barriers to the implementation of MECC in sites where MECC has been implemented to varied degrees of success from the perspective of healthcare professionals and staff responsible for supporting the implementation of MECC.

***Design***

A qualitative study will be conducted with healthcare professionals and those responsible for or supporting MECC implementation in sites which are at different stages of MECC implementation.

***Methods***

Through the survey responses (study 1.1), and consultation with HSE MECC Implementation Group, the research team will aim to identify four sites that are at different stages of MECC implementation. In Ireland, health services are delivered in the community through specific geographical catchments called Community Health Organisations and acute services are delivered through Hospitals aligned together in groups called Hospital Groups. We will seek to select two Community Health Organisations and two Hospital Groups exemplar sites for inclusion in the study. Semi-structured interviews will be conducted with healthcare professionals from these sites who have consented to be contacted for this study during the survey in study 1.1 or via e-mail invitations distributed by a MECC liaison in each study site. If site-based recruitment of sufficient healthcare professionals (i.e. six staff members per site) is not possible, we will recruit healthcare professionals more broadly through directly inviting participants from any HSE site who have consented to be contacted for the qualitative study while participating in the survey study. We will also invite non-clinical staff (e.g. MECC trainers and Health Promotion and Improvement staff) who have responsibility for supporting the implementation of MECC to participate to enhance our understanding of the organisational-level enablers of and barriers to MECC implementation. Non-clinical staff will be invited to the study via e-mail from the HSE MECC team.

We will aim to recruit approximately 24 healthcare professionals and non-clinical staff to the study, recruiting approximately six staff per site. This is in line with a previous qualitative study of healthcare professionals’ barriers and enablers to MECC delivery in the UK ( Keyworth *et al.*, 2019). We will use the survey data and information held by the MECC team on implementation progress at MECC exemplar sites to purposively recruit individuals in different health care professional roles, of different ages and gender, from different health service settings, and those with varied levels of engagement with MECC training and delivery. Final recruitment figures will be determined by examining data adequacy ( Vasileiou *et al.*, 2018) and sample sufficiency for maximising variation in participant demographic variables, study site characteristics and participants’ level of engagement with MECC training and delivery.

Within the semi-structured interviews, healthcare professionals and non-clinical staff will be asked to discuss their experiences of what has enabled them to implement MECC within their site, any barriers they experienced to implementing MECC and any strategies used to overcome implementation barriers. Online or telephone interviews will be conduct by OM, a postdoctoral researcher in health psychology. An interview schedule has been developed to explore the 14 constructs of the Theoretical Domains Framework ( Cane *et al.*, 2012; Michie *et al.*, 2005). The interview schedule was developed with reference to previous literature ( Keyworth *et al.*, 2019; Nelson *et al.*, 2013) and in consultation with the Study Management Team, MECC Implementation Group and the Health Psychology Public Involvement Panel. The interview schedule is publicly available via the Open Science Framework ( Meade *et al.*, 2021). Interviews/focus groups will be recorded and transcribed verbatim. Participants will be offered a €20 voucher as a thank you for their contribution to the study.

***Analysis***

A deductive framework analysis approach ( Gale *et al.*, 2013) will be used to code interview content in relation to the Theoretical Domains Framework ( Cane *et al.*, 2012; Michie *et al.*, 2005).

### WP2 - Patient attitudes and experiences

**Work package overview**

WP2 will focus on patient-level factors related to the implementation of MECC in routine practice. Patient attitudes towards, and experiences of, discussing behavioural risk factors and receiving MECC interventions from healthcare professionals in routine clinical practice will be investigated in a qualitative interview study (study 2.1).

**Study 2.1 A qualitative interview study to examine patient attitudes towards, and experiences of receiving MECC brief interventions**

***Aim***

To understand patient attitudes towards and experiences of receiving MECC brief interventions.

***Design***

A qualitative study will be conducted with patients who have received MECC interventions in community and hospital-based settings.

***Methods***

Semi-structured telephone or online interviews will be carried out by OM (postdoctoral researcher in health psychology) with adult patients who have received MECC interventions in HSE sites where MECC has been implemented. It is anticipated that we will recruit patients from at least one community-based and one hospital-based site. An interview schedule will be developed in line with previous literature ( Elwell *et al.*, 2013; Keyworth *et al.*, 2020a) and in consultation with the Study Management Team, Health Psychology Public Advisory Panel and MECC Implementation Group. Patient attitudes and experiences will be explored broadly and prompts (e.g. the MECC 5As model and the MECC Client Record) will be presented to participants in order to elicit their experiences and preferences in relation to the process of receiving MECC brief interventions. Interviews will be recorded and transcribed verbatim. Participants will be offered a €20 voucher as a thank you for their contribution to the study.

We will use purposive sampling to recruit patients who have received MECC interventions. We will ask healthcare professionals at participating sites to give study information leaflets to patients who they have delivered a MECC intervention to. The information leaflet will invite patients to contact the research team and participate in the study. We will also display study posters in waiting rooms of relevant study sites. We will aim to recruit approximately 24 patients to the interview study, consistent with a previous study of patient perceptions of GP’s delivering brief interventions in the UK ( Keyworth *et al.*, 2020a). We will recruit patients from at least two sites, one hospital-based and one community based. If possible, we will sample participants who differ in terms of gender, age profiles, the type of healthcare professional they received a MECC intervention from and the healthcare setting they received a MECC intervention in. Final recruitment figures will be determined by examining data adequacy ( Vasileiou *et al.*, 2018) and sample sufficiency in relation to maximising variation in participant demographic variables and study site characteristics.

***Analysis***

Inductive thematic analysis will be used to describe, organise and report patterns in the data in relation to patients’ experiences and preferences for discussing health behavioural risk factors with healthcare professionals and receiving MECC brief interventions. Thematic analysis guidelines ( Braun & Clarke, 2006; Braun & Clarke, 2019) will be followed and a realist approach to the data will be taken, whereby interview data will be analysed at a sematic level.

### WP3 – Development of a collaborative implementation strategy

**Work package overview**

In WP3 the research team will use the Behaviour Change Wheel ( Michie *et al.*, 2011; Michie *et al.*, 2014) approach, a systematic approach to intervention development, to combine research evidence from WP1 and WP2 to develop implementation strategy options to optimise and scale-up MECC. A consensus workshop with all stakeholders (The Health Psychology Public Advisory Panel, the Study Management Team, the Study Steering Committee, the MECC Implementation Group, healthcare professionals and Health Promotion and Improvement staff) will be used to achieve consensus on a blueprint for the enhanced implementation of MECC.

**Study 3.1 Development of testable MECC implementation strategies through the use of the Behaviour Change Wheel approach**

***Aim***

To collaboratively develop with key stakeholders a list of implementation strategy options for the improved implementation of MECC in practice, using the Behaviour Change Wheel approach.

***Methods***

In this study we will consolidate findings from studies 1.1, 1.2, 2.1 and the international literature using the Behaviour Change Wheel ( Michie *et al.*, 2011; Michie *et al.*, 2014) to develop implementation strategy options for future MECC implementation. The Behaviour Change Wheel follows three phases: understanding the problem, identifying the type of strategy that might be useful, and identifying specific content.

***Analysis***

Through analysis of studies 1.1, 1.2 and 2.1, review of the international literature and consultation with our stakeholders groups (Health Psychology Public Advisory Panel, Study Steering Committee, and MECC Implementation Group) the Study Management team will identify priority healthcare professional behaviours which are key to the successful implementation of MECC. Potential relevant types of strategies (labelled as functions within the Behaviour Change Wheel approach) and behaviour change techniques that could be used to target these key implementation behaviours will be identified using the Behaviour Change Wheel. These functions and techniques will be evaluated using the APEASE criteria (Acceptability, Practicability, Effectiveness/cost-effectiveness, Affordability, Safety/side-effects, Equity) ( Michie *et al.*, 2014) in consultation with the MECC Implementation Group, Study Steering Committee and our PPI Advisory Panel to decide on a list of appropriate implementation strategy options for the Irish healthcare context. The Template for Intervention Description and Replication (TIDieR) checklist ( Hoffmann *et al.*, 2014) will be used to specify details of intervention strategy elements including who needs to do what, how and where in order to implement the strategy options listed.

**Study 3.2 Consensus workshop to achieve consensus on the MECC implementation strategy and implementation blueprint**

***Aim***

To present implementation strategy options identified in study 3.1 to key stakeholders, for refinement and to achieve consensus on an implementation blueprint for the national implementation of MECC.

***Design***

A consensus-based workshop will be conducted with key stakeholders.

***Methods***

Research and knowledge user members of the research team, representatives of the MECC Implementation Group and relevant senior HSE staff, healthcare professionals, Health Promotion and Improvement staff and members of the Health Psychology Public Advisory Panel will be invited to attend a one day stakeholder workshop. Approximately 20 participants will be included in total, which is in keeping with previous consensus-based workshops (e.g. Walsh *et al.*, 2018). The workshop will be facilitated by an independent expert in health services implementation research and consensus methods.

During the first half of the workshop an update on the MECC programme will be provided in addition to a summary of the research programme findings from studies 1.1, 1.2, 2.1 and 3.1. An accessible summary of the study findings will also be provided to stakeholders prior to the meeting. The implementation strategy options identified in study 3.1 will then be presented to key stakeholders. We will use an adapted version of a consensus building process used previously in the development of an intervention to improve outcomes for young people with diabetes ( Walsh *et al.*, 2018). All participants will be asked to rate each strategy on a five-point Likert scale (1 = very low impact, 5 = very high impact) according to the potential impact each strategy might have on enhancing the implementation of MECC by healthcare professionals. Participants will also be asked to rate how feasible it would be to implement each strategy in practice on a five-point Likert scale (1 = very low feasibility, 5 = very high feasibility).

During the second half of the workshop, the top implementation strategies (strategies which received above average scores for both impact and feasibility in the first half of the workshop) will be presented back to key stakeholders. We will then work with stakeholders to develop a formal implementation blueprint for these strategies. As described in the Expert Recommendations for Implementing Change (ERIC), an implementation blueprint should include all agreed implementation strategies and specific goals of how these strategies will be enacted ( Powell *et al.*, 2015). As per ERIC guidance, the blueprint will also outline 1) aim/purpose of the implementation; 2) scope of the change (e.g., what organizational units are affected); 3) timeframe and milestones; and 4) appropriate performance/progress measures.

Through small group brainstorming, and larger group discussion, we will also work with stakeholders to identify how this blueprint may be used, evaluated and updated over time, and who is best placed to lead the on-going implementation beyond the lifespan of the current research programme. An action-oriented, policy brief of key findings will be prepared and a dissemination strategy agreed with research and knowledge users within the team, to ensure maximum penetration and engagement.

***Analysis***

During the first half of the meeting we will collate participant ratings of the potential impact and feasibility of strategies to enhance MECC implementation using descriptive statistics. Implementation strategies that received above average scores for both impact and feasibility will be brought forward to the second part of the workshop to be included in the implementation blueprint. During the second half of the meeting, we will use ERIC guidance to develop the formal implementation blueprint for the national implementation of MECC.

### Dissemination and reporting

Throughout the course of the research programme, the in-progress findings will be presented at national and international conferences which have both academic and healthcare professional foci and presentation of findings at conferences will be shared between knowledge user and academic researcher team members. International dissemination will be enhanced through an invited workshop at the International Behavioural Trials Network conference 2022. A research programme website and a social media account (@makingmeccwork) have been developed to maximise research dissemination. Lay summaries of all study findings will be produced with support from our Health Psychology Public Advisory Panel and will be posted on the study website. Lay summaries will also be e-mailed/posted to all study participants who wish to receive a copy of our study findings.

We will use relevant reporting guidelines to enhance the completeness of our academic publications including the STrengthening the Reporting of OBservational studies in Epidemiology (STROBE) Statement ( von Elm *et al.*, 2007) for our survey study (1.1), the COnsolidated criteria for REporting Qualitative research (COREQ) checklist ( Tong *et al.*, 2007) for our qualitative studies (1.2, 2.1), the CREDES guidelines ( Jünger *et al.*, 2017) for our consensus study (3.2) and the Guidance for Reporting Involvement of Patients and the Public Version 2 (GRIPP2) checklist ( Staniszewska *et al.*, 2011) for reporting on patient and public involvement within each study.

### Data management and sharing

Identifying data will be stored on a password-protected computer accessible only to the research team. Data will be anonymised prior to analysis. Interview recordings will be destroyed once recordings have been transcribed and anonymised. All research shall be conducted in line with the Data Protection Acts 1998 and 2003, the General Data Protection Regulation (GDPR) and all applicable National and EU regulations.

Study outputs, data and analysis protocols will be made openly accessible via public repositories and/or by request. The exception to this may be where participants do not consent to data sharing or it is not possible to protect participants’ identities despite attempts at anonymization (e.g. in the case of interview transcripts). Any data to be shared publicly will be anonymised in advance and GDPR will be adhered to.

## Ethical considerations

The research programme will involve adult participants who will give fully informed consent to participate. It will be made clear to potential participants that they are under no obligation to participate and, even if they do agree to be part of this study, they may withdraw at any time if they feel uncomfortable. Healthcare professionals will be assured that their clinical practice is not being evaluated. Patients will be assured that their participation will not be disclosed to healthcare professionals and their decision to take part or not take part will not affect their care in any way. Ethical approval will be sought for the research through the National University of Ireland Galway Ethics Committee and relevant HSE Ethics Committees.

## Study status

WP1 Study 1.1. – Ethical approval has been gained (NUI Galway – Ref R20.Jun.16). Recruitment is complete.

WP1 Study 1.2 – Ethical approval gained (NUI Galway – Ref 2020.08.012). Recruitment is currently underway.

WP 2 Study 2.1 – This study has not started yet.

WP3 Study 3.1 – This study has not started yet

WP3 Study 3.2 – This study has not started yet.

## Conclusion

In Ireland, there are approximately 30 million contacts within the health service each year ( Health Service Executive, 2016). MECC has the potential for significant impact on the prevention and management of chronic disease by integrating brief interventions to support health behaviour change into these millions of contacts that healthcare professionals have with members of the public each year. Our research partnership brings together academic researchers with implementation and health psychology expertise, knowledge users with strategic and context-specific knowledge, patient and public contributors and healthcare professionals to optimise the implementation of the MECC in the Irish Health Service. From WP1, the research process and outputs with healthcare professionals and staff with a role in MECC implementation will allow lessons learnt from initial MECC implementation to inform further roll out at a national scale. From WP2, the engagement of patients and the exploration of their views will ensure the public voice informs the development of the MECC implementation strategy, a relatively unexplored aspect in the existing literature. Finally, WP3 will use a structured approach to combine evidence from WP1 and 2, results from an existing international literature review ( Keyworth *et al.*, 2020b)) and an expert stakeholder consensus workshop to collaboratively agree priority strategy options and an implementation blueprint for the optimisation and scaling up of the MECC programme. The outputs from this programme of research will have a clear impact on the delivery of MECC by developing an evidence base and implementation blueprint to support the integration of brief behaviour change intervention into the Irish health system. It will also build on the limited international literature on the implementation of behaviour change programmes that take a standardised national approach to brief intervention training and implementation support. 

## Data availability

### Underlying data

No data are associated with this article.

### Extended data

Open Science Framework: Enhancing the implementation of the Making Every Contact Count brief behavioural intervention programme in Ireland - the 'Making MECC Work' project, https://doi.org/10.17605/OSF.IO/EF5SM ( Meade *et al.*, 2021).

This project contains the following extended data:

- Workpackage 1 Study 1.1 MECC survey .pdf
- Workpackage 1 Study 1.1 MECC Survey Sample Size Calculation.pdf
- Workpackage 1 Study 1.2 Interview schedule.pdf

Data are available under the terms of the Creative Commons Zero "No rights reserved" data waiver (CC0 1.0 Public domain dedication).
