# Supplementary material for: Enhancing the implementation of the Making Every Contact Count brief behavioural intervention programme in Ireland: protocol for the Making MECC Work research programme
Source: HRB Open Res. 2022 Jan 18;5:6. [Version 1] doi: 10.12688/hrbopenres.13481.1 (PMC8847722; doi:10.12688/hrbopenres.13481.1)
Supplement: Peer review history

# Peer review: Reviewer response for version 1

**DOI:** 10.21956/hrbopenres.14698.r31268

**Published:** 2022-02-15

**Authors:** Vivien Swanson

**Affiliations:** 1 Psychology Division, University of Stirling, Stirling, UK

This article is a study protocol for a research programme that aims to evaluate and inform the subsequent implementation of the Making Every Contact Count (MECC) Programme in Ireland. MECC focuses on using communication and behaviour change skills to maximise healthcare professionals' opportunities to engage with service users in a range of settings to support a range of health behaviours. There are three work programmes engaging staff, service users and external stakeholders using mixed methods. The first stage, a quantitative survey of MECC use by healthcare professionals, is underway. 

 Brief behaviour change interventions have been developed and used internationally, generally focusing on specific health conditions or behaviours, and MECC interventions have gained international traction as being accessible and helpful for service users and providers. 

 The article introduction effectively scopes out what is known already about the barriers and facilitators to implementation. We also learn that the MECC programme was first introduced in Ireland in 2019, starting with 60 implementer sites. At this point, it would be helpful to clarify what uptake of the programme in Ireland has been to date (in terms of geographic spread and local engagement by health and care professionals), including engagement in different stages of training and the timescale for future roll-out plans.  The existing MECC framework used to inform roll-out appears rigorous in terms of mapping, implementation, training approach and monitoring, so it is not very clear what this current evaluation will add, how and when. Clarifying this would help to make a stronger case for supporting the very rigorous evaluation and assessment processes planned in the protocol. 

 The overall planned research design, methodology and data analysis plans are appropriate and theory-informed and the work programmes fit together well to provide the information needed for enhancing implementation.

 In relation to the assessment of existing MECC training and healthcare professionals' use of interventions, our own work has suggested this may be difficult for some to recall given the three-year period during which training has been delivered and the intervening period of the pandemic (when many NHS staff were redeployed). It may be that the use of MECC may have been sporadic and atypical during this period. I wonder if using specific time-based prompts would be useful. It also occurred to me that many interactions will have been remote during this time and it would be helpful to capture differences between remote and face-to-face working. 

 A key strength of this proposed programme is the collaborative involvement of service users in all phases of the work. Service providers don't always assess demand, need for interventions, or consider optimal forms of delivery, which is bound to ultimately affect uptake. This work, including the semi-structured interviews of service users, is therefore important. I did wonder about the reliability of data including service users' awareness of the use of MECC interventions and if they are specifically labelled as such in different healthcare contexts. It may be challenging to separate out the content of the intervention from the consultation style of the person or multidisciplinary team delivering it in some cases, particularly where it is delivered opportunistically or as part of an appointment for a specific health condition. 

 In relation to WP3, it would be great if consideration could be given to the form of delivery of MECC interventions, as well as content and contextual factors. Our own experience with the delivery of behaviour change training programmes for health professionals over the past three years has suggested that the pandemic has had a high level of impact on staff's ability to engage with training, and also to be able to implement new skills learned in training due to time and exceptional workforce demands. Future implementation strategies for the MECC programme may benefit from explicitly considering staff's need for additional training, support, coaching and mentoring, and the managerial and workforce resources to develop, use and maintain these new skills and sets of competencies going forward.

Is the study design appropriate for the research question?

Yes

Is the rationale for, and objectives of, the study clearly described?

Yes

Are sufficient details of the methods provided to allow replication by others?

Yes

Are the datasets clearly presented in a useable and accessible format?

Not applicable

Reviewer Expertise:

Health psychology, including expertise in the delivery of behaviour change interventions and training health professionals. Diabetes and maternal health and well-being are main topic areas.

I confirm that I have read this submission and believe that I have an appropriate level of expertise to confirm that it is of an acceptable scientific standard.

# Peer review: Reviewer response for version 1

**DOI:** 10.21956/hrbopenres.14698.r31267

**Published:** 2022-01-25

**Authors:** Edurne Zabaleta-del-Olmo

**Affiliations:** 1 Fundació Institut Universitari per a la recerca a l'Atenció Primària de Salut Jordi Gol i Gurina (IDIAPJGol), Barcelona, Spain; 2 Universitat de Girona, Girona, Spain; 3 Institut Català de la Salut, Barcelona, Spain

This protocol describes the design of a study aimed at assessing the barriers and enablers for implementing a national programme for behaviour change in acute and community settings in Ireland and designing an implementation strategy in line with the results of this assessment. The research topic is crucial and necessary as the systematic integration of health promotion interventions into the routine practice of healthcare professionals can translate into relevant health outcomes at different levels, at the citizen level, at the societal level, and at the health system level. The protocol is very well written and adequately describes the essential details of the research. However, I believe some of the issues detailed below could be improved.

- In studies 1.1. and 1.2., I have a question about the selection criteria for participants. Why will only healthcare professionals who completed the eLearning training participate in the survey? Perhaps it would be interesting to know the perspective of those healthcare professionals who did not complete the training or did not start it. Understanding the barriers perceived by these health professionals is a very relevant issue from my point of view.
- The questionnaire used in study 1.1. is based on the Theoretical Domains Framework (TDF) and different expert committees reviewed its content validity. The researchers say that they will analyse its structural validity using confirmatory factor analysis and that based on these results they will eliminate items with low factor loadings. Therefore, I understand that the researchers consider the questionnaire to be based on a reflective model and they will carry out a pilot test to develop a final version. However, the protocol does not describe how this pilot test will be carried out in the target population and whether, in addition to statistical criteria, issues such as comprehensibility, relevance, and comprehensiveness of the items will be evaluated by the target population. On the other hand, I think it would be interesting to consider more methods to support the decision to remove items from the questionnaire and not base it solely on the analysis of its structure (item discrimination index or missing cases, for example). Furthermore, it would be interesting to analyse issues related to this interpretability (distribution of scores, percentages of missing items, ceiling, and floor effects) and feasibility (completion time, ease of administration, required equipment). The assessments of these issues can also help decide which items are the most important for the study. Furthermore, I believe that analyses of the construct validity of the questionnaire could be carried out by assessing the relationship of its scores to the extent of the actual recording of MECC interventions in electronic health records, for example. The hypothesis would be that healthcare professionals who have recorded a higher number of interventions would score higher on the questionnaire.
- The overall theoretical framework of the study is the TDF, an integrative theoretical framework specifically designed to assess implementation in behaviour change and would therefore be suitable for carrying out the study. However, in my opinion, the rationale for choosing this particular theoretical approach is not sufficiently justified in the protocol. On the other hand, given the complex nature of the implementation of the MECC, I think it could be interesting to combine this framework with other possible frameworks such as the Consolidated Framework for Implementation Research (CFIR). This combination could help improve the understanding of the determinants and outcomes of programme implementation.
- The researchers describe that the MECC programme was implemented in “exemplar healthcare sites” in 2019. It would be interesting to explain the concept "exemplar" and what characteristics these sites had that made them have this status. It is important to assess the applicability of the study results to other healthcare sites.

Is the study design appropriate for the research question?

Yes

Is the rationale for, and objectives of, the study clearly described?

Yes

Are sufficient details of the methods provided to allow replication by others?

Partly

Are the datasets clearly presented in a useable and accessible format?

Not applicable

Reviewer Expertise:

Health Promotion; Implementation Research; Nursing; Primary Health Care; Psychometrics; Systematic Reviews

I confirm that I have read this submission and believe that I have an appropriate level of expertise to confirm that it is of an acceptable scientific standard.
